# Early immune modulation by single-agent trastuzumab as a marker of trastuzumab benefit

**DOI:** 10.1038/s41416-018-0318-0

**Published:** 2018-11-27

**Authors:** Tiziana Triulzi, Viola Regondi, Loris De Cecco, Maria Rosa Cappelletti, Martina Di Modica, Biagio Paolini, Pier Luigi Lollini, Serena Di Cosimo, Lucia Sfondrini, Daniele Generali, Elda Tagliabue

**Affiliations:** 1 Molecular Targeting Unit, Department of Research, Fondazione IRCCS Istituto Nazionale dei Tumori di Milano, Milano, Italy; 2 Platform of Integrated Biology, Department of Applied Research and Technology Development, Fondazione IRCCS Istituto Nazionale dei Tumori di Milano, Milano, Italy; 3 U.O. Multidisciplinare di Patologia Mammaria e Ricerca Traslazionale, Azienda Socio-Sanitaria Territoriale di Cremona, Cremona, Italy; 4 Anatomic Pathology A Unit, Department of Pathology, Fondazione IRCCS Istituto Nazionale dei Tumori di Milano, Milano, Italy; 5 Laboratory of Immunology and Biology of Metastases, Department of Experimental, Diagnostic and Specialty Medicine, University of Bologna, Bologna, Italy; 6 Department of Applied Research and Technology Development, Fondazione IRCCS Istituto Nazionale dei Tumori di Milano, Milano, Italy; 7 Dipartimento di Scienze Biomediche per la Salute, Università degli Studi di Milano, Milano, Italy; 8 Dipartimento Universitario Clinico di Scienze Mediche, Chirurgiche e della Salute, Università degli Studi di Trieste, Trieste, Italy

**Keywords:** Breast cancer, Gene expression, Predictive markers, Immunotherapy

## Abstract

**Background:**

Optimising the selection of HER2-targeted regimens by identifying subsets of HER2-positive breast cancer (BC) patients who need more or less therapy remains challenging. We analysed BC samples before and after treatment with 1 cycle of trastuzumab according to the response to trastuzumab.

**Methods:**

Gene expression profiles of pre- and post-treatment tumour samples from 17 HER2-positive BC patients were analysed on the Illumina platform. Tumour-associated immune pathways and blood counts were analysed with regard to the response to trastuzumab. HER2-positive murine models with differential responses to trastuzumab were used to reproduce and better characterise these data.

**Results:**

Patients who responded to single-agent trastuzumab had basal tumour biopsies that were enriched in immune pathways, particularly the MHC-II metagene. One cycle of trastuzumab modulated the expression levels of MHC-II genes, which increased in patients who had a complete response on treatment with trastuzumab and chemotherapy. Trastuzumab increased the MHC-II-positive cell population, primarily macrophages, only in the tumour microenvironment of responsive mice. In patients who benefited from complete trastuzumab therapy and in mice that harboured responsive tumours circulating neutrophil levels declined, but this cell subset rose in nonresponsive tumours.

**Conclusions:**

Short treatment with trastuzumab induces local and systemic immunomodulation that is associated with clinical outcomes.

## BACKGROUND

The HER2 receptor is overexpressed in approximately 20% of breast cancers (BCs) and has historically been associated with aggressive disease and a poor prognosis. Treatment with trastuzumab and chemotherapy has dramatically changed the prognosis of early and advanced HER2-positive (HER2+) BC patients.^1^ More recently, the introduction and availability of other HER2-targeting agents offer clinicians the ability to provide prolonged inhibition of HER2 signalling across multiple lines of treatment, and the dual HER2 blockade approach has demonstrated superior activity over trastuzumab alone. As more potential therapies appear over the horizon, advancements in biomarker discovery will be critical in optimising treatment selection and providing personalised therapy for patients. Particularly, it remains unknown which patients benefit from single-agent trastuzumab and those who require instead the dual blockade upfront. Many efforts have been made toward identifying biomarkers that predict a benefit from trastuzumab: HER2 addiction and immune features have demonstrated the best predictive ability in several trials (reviewed in ref. ^2^), but HER2 expression in the primary tumour remains the only marker that is used in clinical practice.

Preclinical studies have reported that trastuzumab has cytostatic (e.g., inhibition of HER2 signalling, extracellular domain shedding, and tumour angiogenesis) and cytotoxic activities [antibody-dependent cell-mediated cytotoxicity (ADCC), antibody-dependent cell-mediated phagocytosis (ADCP), and inhibition of DNA repair].^3^ In the neoadjuvant setting, the decrease in tumour burden, or pathological complete response (pCR), is likely to depend on the cytotoxic activity of trastuzumab.^4^ Accordingly, increasing evidence has shown that the therapeutic activity of trastuzumab relies on the innate and adaptive immune systems, and the significant pre-existing infiltration of BCs by immune cells has been associated with pCR in several trials (reviewed in ref. ^5^). Trastuzumab also positively regulates infiltration by lymphoid cells in tumour tissues,^6–8^ supporting that its clinical activity is based on its immunostimulatory effects.^9^

In this study, we analysed the gene expression profile of tumour biopsies from the TRastuzumab UPfront in HER2+ locally advanced BC (TRUP) window-of-opportunity trial^10^ before and after brief exposure to trastuzumab. We performed an exploratory analysis of the molecular features that were associated with the clinical activity of short-term trastuzumab monotherapy, monitoring changes in immune expression on treatment as markers of trastuzumab activity.

## Materials and methods

### Patients

The 17 tumours that we profiled were obtained from BC patients in the TRUP window-of-opportunity trial,^10^ which comprised 28 locally advanced primary BCs that were diagnosed by incisional biopsy at A.O. Istituti Ospitalieri di Cremona. In the TRUP study, patients received 1 cycle of trastuzumab alone, followed by 4 cycles of trastuzumab and paclitaxel, until definitive surgery. As per the protocol, after single-agent trastuzumab (21 days), assessment of tumour response and research tumour biopsy (Tru-cut biopsy) were performed. Responses at the Tru-cut biopsy were available for all but one patient. Blood test results (i.e., blood cell count/μl) at baseline and at the Tru-cut biopsy were retrieved and analysed. All procedures were performed in accordance with the Helsinki Declaration. The biospecimens that were examined consisted of leftover material from samples that had been collected during standard surgical and medical procedures at A.O. Istituti Ospitalieri di Cremona. Samples were donated by patients to the Institutional BioBank for research purposes, and aliquots were designated for this study after approval by the institutional review board and a specific request to the independent ethical committee ‘Comitato Etico Val Padana’.

The response to trastuzumab after 21 days was calculated as % reduction in clinical dimensions or in the number of Ki67-positive cells at day 21 compared with baseline. Patients who experienced a decrease of at least 20% in tumour volume or at least 50% in the number of Ki67-positive cells were scored as responders. The response rate (RR) to trastuzumab and chemotherapy was calculated as the ratio of the difference between the initial clinical dimensions and final pathological dimensions to the initial clinical dimensions, and responders were selected, based on the cut-off RR in the original study (RR = 70%).^10^

### Gene expression analysis

RNA was extracted from frozen incisional biopsies of the TRUP cohort using the miRNeasy MINI KIT (Qiagen) according to the manufacturer’s protocol. RNA quality was checked in a preanalytical screen by RT-qPCR, as described.^11^ Gene expression profiles were generated by whole-genome DASL (cDNA-mediated annealing, selection, extension, and ligation) assay using HumanHT12_v4 BeadChips (Illumina), according to a standard protocol. The Illumina BeadArray Reader was used to scan the arrays. Illumina BeadScan was used to acquire the images and recover the primary data. The data were quantile-normalised using BeadStudio. The BeadChips covered over 29,000 annotated genes that were derived from RefSeq (Build 36.2, Release 38), and after a filter step, a data matrix that contained 17 matched samples was generated. The data were deposited into the Gene Expression Omnibus repository (accession number GSE114082).

### Bioinformatics analysis

Gene set enrichment analysis was performed for Reactome pathways using GSEA v2.0.13.^12^ Genes that were represented by more than 1 probe were collapsed to the probe with the maximum value using the Collapse Dataset tool. Gene set permutation type was applied 1000 times, and gene set enrichment was considered to be significant at a false discovery rate (FDR) of <10%.

Immune metagenes were determined per Rody et al.^13^ The 569 Affymetrix probe sets were first mapped using the Illumina platform, and of  the 7 immune metagenes, the IgG metagene was excluded due to low gene number. The average log-transformed expression of the genes that belonged to each metagene was calculated. Cluster analysis of Rody’s metagenes was performed using Cluster 3.0 by K-mean clustering (*n* = 3) and visualised using TreeView. To estimate the abundance of immune cell types in TRUP tumours, we applied the CIBERSORT tool to our dataset using the LM22 signature.^14^

### Immunofluorescence and immunohistochemistry

IHC was performed on formalin-fixed paraffin-embedded (FFPE) tissue. Slides were deparaffinised in xylol and serially rehydrated, and after antigen retrieval at 96 °C for 6 min using 10 mM citrate buffer, pH 6.0, the samples were stained with mouse monoclonal anti-human HLA-DR/DP/DQ/DX (clone CR3/43, 1:500, Santa Cruz Biotechnology). Immunoreactions were visualised using streptavidin-biotin-peroxidase (Thermo Fisher Scientific) and the DAB Chromogen System (Dako Agilent Technologies) and counterstained with Carazzi haematoxylin.

For double immunofluorescence, the sections were subjected to 2 sequential rounds of single-marker immunostaining, as reported.^15^ The samples were incubated with mouse anti-human CD68 (clone KP1, 1:100, Novus Biologicals) and then with Alexa Fluor 488-conjugated secondary antibodies. Subsequently, mouse anti-human HLA-DR/DP/DQ/DX that was labelled using the Zenon Alexa Fluor-546 mouse IgG labelling kit (Molecular Probes) was applied. The samples were imaged under a Leica TCS SP8 X confocal laser scanning microscope (Leica Microsystems GmbH). The fluorochrome was excited by a pulsed supercontinuum white-light laser (470–670 nm; 1-nm tuning step size). Specifically, CD68 was excited by a 499-nm-laser line and detected from 504 to 561 nm; HLA-DR/DP/DQ/DX was excited by a 557-nm laser line and detected from 562 to 642 nm; and nuclei were visualised using DAPI, which was excited with a 405-nm diode laser and detected from 422 to 488 nm. The images were acquired in 512 × 512 scan format using an HC PL APO 40 × /1.3 CS2 oil immersion objective and a pinhole that was set to 1 airy unit. The data were analysed using Leica LAS X rel. 3.1.1 (Leica Microsystems GmbH). Cells were counted manually in 3 40X fields for each slide.

### In vivo experiments

Six- to eight-week-old female FVB mice (Charles River) were maintained in laminar flow rooms at constant temperature and humidity, with food and water given ad libitum. The experimental protocols for the animal studies were approved by the Ethics Committee for Animal Experimentation (OPBA) of Fondazione IRCCS Istituto Nazionale dei Tumori di Milano according to institutional guidelines.

We injected the FVB mice with syngeneic MI6 or WTHER2_1 cells (1 × 10^6^) into the mammary fat pad (m.f.p.), as described.^16^ MI6 and WTHER2_1 cell lines were previously established from spontaneous primary mammary carcinomas of FVB-d16HER2 and FVB-huHER2 transgenic female mice, respectively.^16,17^ Both the MI6 and WTHER2_1 m.f.p. models in FVB mice strictly recapitulated morphologic, aggressive and trastuzumab-responsive features of spontaneous primary mammary tumours.^16^ When the tumours reached a mean volume of about 100 mm^3^, the animals were randomised into 2 groups (*n* = 4/group) to receive 2 intraperitoneal injections of 5 mg/kg trastuzumab or saline on days 1 and 4 after randomisation. Tumours were harvested and analysed 7 days after the treatment outset.

### Flow cytometry

The cells that were derived from the digestion of the murine mammary carcinomas with collagenase (300 U/ml) and hyaluronidase (100 U/ml) (StemCell Technologies) were analysed by direct immunofluorescence (IF) as described.^18^ Cells were stained with the following antibodies: CD45APCeFluor780 (30-F11, eBioscience), CD11bPE (MI/70, BD Bioscience), F4/80FITC (BM8, eBioscience), and MHCIIAPC (M5/114.15.2, Miltenyi Biotec). Purified rat monoclonal anti-mouse CD16/CD32 (eBioscience) was used to prevent nonspecific binding to mouse Fc receptors. The samples were analysed by gating on live cells after doublet exclusion on a FACSCanto system (BD Bioscience) using FlowJo (Tree Star Inc).

### Statistical analysis

Differences between groups were determined by two-tailed student’s *t*-test. Associations between categorical variables were analysed by Fisher’s exact test, and correlations between continuous variables were examined by Spearman correlation analysis. A two-sided *p*-value of <0.05 was considered to be significant. The statistical analysis was performed using the GraphPad Prism 5.01 package (GraphPad).

## Results

### Molecular features associated with trastuzumab antitumour activity

To identify the molecular features that are associated with trastuzumab activity in the clinical setting, we analysed tumour biopsies from patients with locally advanced primary HER2+ BC at diagnosis who underwent brief exposure to trastuzumab monotherapy.^10^ From a cohort of 28 patients, RNA samples with sufficient quality and quantity for gene expression profiling were available for matched pretreatment and 21-day samples (Tru-cut samples) for 17 subjects.

We compared the gene expression profiles of pretreatment tumours with regard to the response to short-term trastuzumab. Two types of responses were considered: clinical (C) and Ki67 (K). C response was defined as a reduction in tumour dimensions of at least a 20% between post-therapy day 21 and the clinical dimensions before the administration of trastuzumab. K response was derived from positivity of pretreatment and post-treatment biopsies for a cancer cell proliferation index (Ki67). Patients were considered to be K responders if the number of Ki67-positive cells in the post-treatment biopsy fell by at least 50% compared with the pretreatment biopsy.

**Fig. 1:**
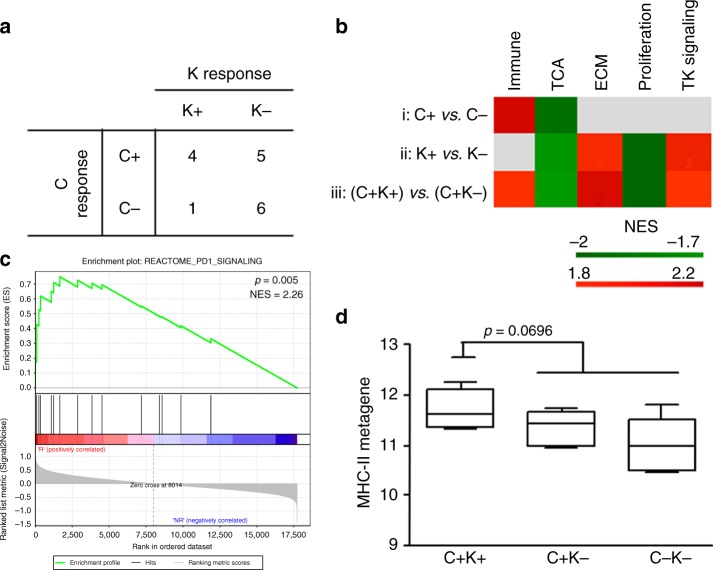
Molecular features of HER2+ BC biopsies associated with trastuzumab cytotoxic and cytostatic activity. **a** Scheme of the number of tumours in each group. C+: responders (patients who experienced a reduction of at least 20% in tumour volume); C−: non-responders. K+: responders (patients who experienced a reduction of at least 50% in the number of Ki67-positive cells in their tumours after treatment with trastuzumab); K−: non-responders. **b** Averaged normalised enrichment score (NES) of pathways in each cluster significantly enriched (FDR <10%) in each comparison. Clusters were visualised using Cytoscape and labelled manually. Grey squares indicate no significant enrichment. **c** GSEA enrichment plots of PD1 gene set in C+ compared with C− tumours. NES, normalised enrichment score. **d** Expression of MHC-II metagene according to tumour partition based on C and K responses. *p*-value by unpaired *t*-test

Figure 1a shows the distribution of responders. Not all cases with a C response experienced a strong reduction in Ki67 levels, whereas most tumours (86%) that were classified C non-responders were also K non-responders. The tumour characteristics by response are shown in Table S1. No differences in tumour size, grade, hormone receptor status, proliferation index, or intrinsic subtype were found between groups.

To examine the differences in gene expression profiles between groups, we performed gene set enrichment analyses of pretreatment biopsies by C and K responses. Compared with clinical unresponsive cases (C−), clinical responsive cases (C+) showed positive and negative enrichment, respectively, in pathways that were related to immune cells and the tricarboxylic acid (TCA) cycle (Fig. 1b and Table S2). PD1 signalling, which primarily comprises MHC-II genes and T cell markers, was the most significantly enriched immune pathway in C responders compared with non-responders (Fig. 1c). In contrast, K response was not associated with immune pathways, but the tumours in K responders (K+) versus non-responders (K−), were positively enriched in pathways that were related to extracellular matrix (ECM) organisation and receptor tyrosine kinase (RTK) signalling and negatively enriched for TCA- and proliferation-related genes (Fig. 1b and Table S2).

Among C responders, we also compared K responders versus K non-responders (C+K+  vs C+K−) and noted enrichment in immune pathways in C+K+ tumours, in addition to pathways found between K+ versus K− patients, indicating that there is greater enrichment in immune genes among clinical responders in tumours that have a robust Ki67 response to trastuzumab versus K-unresponsive cases (Figure S1). Accordingly, a greater response to trastuzumab and chemotherapy at surgery was observed in C+K+ cases than in the other groups (75% C+K+ vs 20% C+K− vs 43% C−K−), suggesting that robust and rapid cytostatic and cytotoxic activity of trastuzumab is predictive of the response to complete preoperative treatment.

### Immune features that are associated with response to trastuzumab

Based on the enrichment of immune genes in trastuzumab responders, the function of the immune system in the cytotoxic activity of trastuzumab, and the performance of immune metagenes in predicting the response to trastuzumab in patients who are treated in the neoadjuvant setting,^5^ we determined the predictive ability of immune-related genes and their modulation in identifying responders to trastuzumab. Based on the presence of MHC-II, interferon-related genes, and T cell markers as leading genes of the immune pathways that were enriched in responders by GSEA (Table S2), we applied to the dataset the Rody immune metagenes that are surrogate markers for the amounts of various immune cell types in BC samples.^13^ Moreover, we used the CIBERSORT tool to measure the relative fractions of immune cell subsets in the samples.

Although we did not observe any differences in the CIBERSORT 22 immune subsets between responders and non-responders (Figure S2), of the 6 Rody metagenes that we applied (MHC-I, MHC-II, HCK, LCK, IFN, and STAT1), C+K+ tumours expressed higher levels of the MHC-II metagene (*p* = 0.0696) than other tumours (Fig. 1d). The lymphocyte-specific kinase (LCK) and haematopoietic cell kinase (HCK) metagenes followed the same trend, albeit insignificantly (Figure S3), as expected, based on the high correlation between LCK and HCK with the MHC-II metagene (*r* = 0.78, *p* = 0.0004 and *r* = 0.84, *p* < 0.0001, respectively), supporting the function of immune genes and cells in the rapid response to trastuzumab. Accordingly, unsupervised clustering of samples at baseline with genes belonging to the Rody’s metagenes showed a partition of C+K+ tumours and a high expression of immune genes in these tumours (Figure S4).

**Fig. 2:**
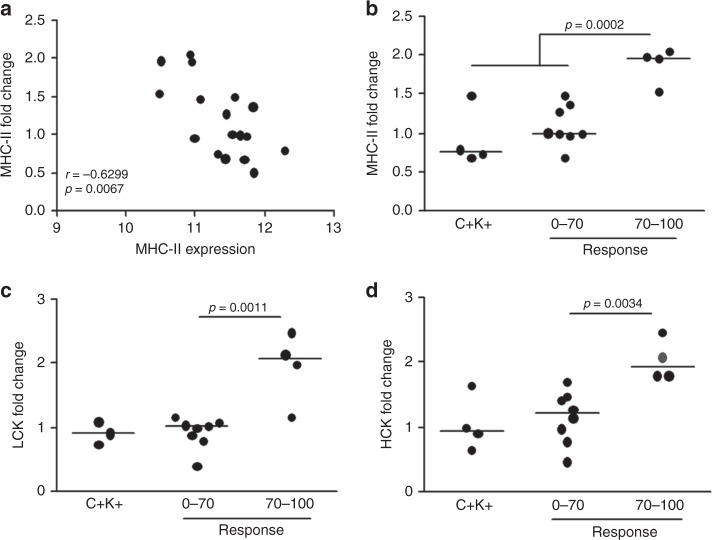
Modification of immune metagene expression by trastuzumab **a** Correlation between MHC-II expression at baseline and its modulation by trastuzumab (fold-change) in all cases of the TRUP cohort. Spearman coefficients and relative *p*-values are shown. **b**–**d** Modulation of MHC-II (**b**), LCK (**c**), and HCK (**d**) metagene expression in post-treatment vs pretreatment biopsies according to response to trastuzumab-based chemotherapy as evaluated at surgery. Fold-change in C+K+ tumours is also shown. *p*-values by unpaired *t-*test

In the analysis of the change in the expression of these metagenes after 1 cycle of trastuzumab, there was a significant inverse correlation between the level of each MHC-II, LCK, and HCK metagene in pretreatment biopsies and their induction by trastuzumab (MHC-II: *r* = −0.63, *p* = 0.0067; LCK: *r* = −0.58, *p* = 0.0140; HCK: *r* = −0.60, *p* = 0.0104) (Fig. 2a and Figure S5). These data indicate that immune cell infiltration is altered in tumours that express low levels of these metagenes (i.e., non-C+K+ tumours) and that trastuzumab effects no such changes in those with high basal expression. Notably, tumours in the TRUP cohort that experienced the greatest increase in these metagenes after trastuzumab treatment belonged to patients who were responsive to complete preoperative trastuzumab-based therapy (Fig. 2b-d). The fold-increase in MHC-II in these tumours was significantly higher than in non-responders and in C+K+ tumours, in which trastuzumab did not significantly alter the immune profile. In a cluster analysis of all tumours these tru-cut biopsies were in the same cluster of C+K+ pretreatment samples, indicating that in these tumours trastuzumab increased immune infiltration up to the level of C+K+ tumours (Figure S4).

The inverse correlation between the up-modulation of immune metagenes by trastuzumab and their basal expression was validated in samples from the GSE76360 dataset (MHC-II: *r* = −0.54, *p* < 0.0001; LCK: *r* = −0.55, *p* < 0.0001; HCK: *r* = −0.48, *p* = 0.0004, Figure S5), although the association with response was unable to be determined due to the unavailability of data on the response to short-term trastuzumab.

### Modulation of MHC-II genes in responsive tumours

**Fig. 3:**
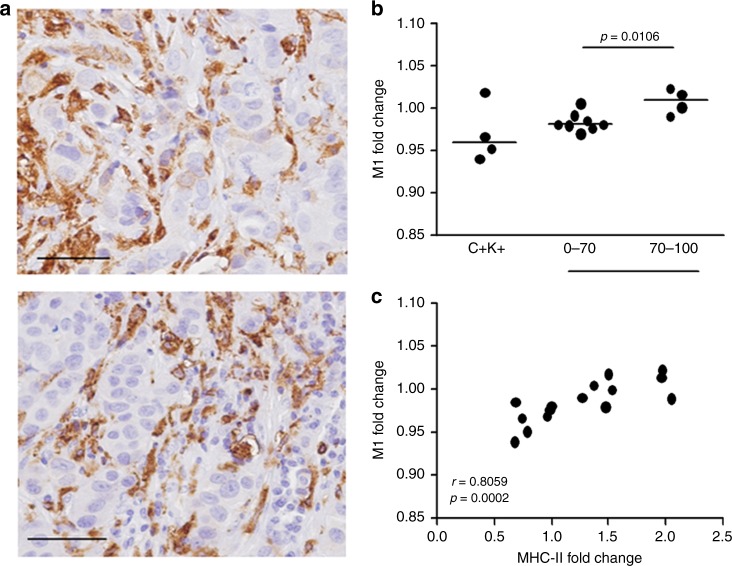
Expression of MHC-II in TRUP tumours. **a** Representative IHC images of MHC-II expression in FFPE tumour biopsies of the TRUP cohort. Scale bars: 50 μm. **b** Change in M1 macrophage fraction as evaluated using the CIBERSORT tool on treatment with trastuzumab in cases of the TRUP cohort according to response to trastuzumab-based chemotherapy, as evaluated at surgery. Fold-change in C+K+ tumours is also shown. *p*-values by unpaired *t*-test. **c** Correlation between changes in MHC-II and M1 (fold-change) induced by trastuzumab in all cases of the TRUP cohort. Spearman coefficients and relative *p*-values are shown

To determine whether the increase in MHC-II after trastuzumab treatment reflects the recruitment and activation of immune cells in the tumour microenvironment or its up-modulation in tumour or stromal cells, we performed IHC of MHC-II using FFPE samples of these biopsies. MHC-II positivity in tumour biopsies was almost exclusive to inflammatory cells of the tumour microenvironment, in which MHC-II-positive lymphocytes and macrophages were detected (Fig. 3a).

The proportion of M1 macrophages, which express MHC-II molecules,^19^ was the only subset that rose significantly after trastuzumab treatment in responsive tumours compared with non-responders by CIBERSORT (Fig. 3b). Accordingly, a direct and robust correlation was observed between the MHC-II metagene and the induction of M1 by trastuzumab, supporting that greater M1 macrophage infiltration on administration of trastuzumab underlies the up-modulation of MHC-II in responsive tumours (Fig. 3c). By immunofluorescence, in certain samples of the TRUP cohort, 70–90% of total CD68+ cells expressed MHC-II molecules (data not shown).

**Fig. 4:**
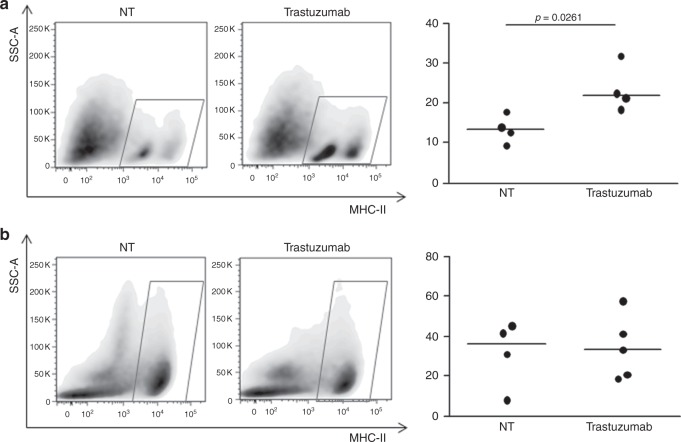
Modulation of MHC-II-positive cells on trastuzumab treatment in HER2+ murine models. **a**, **b** Representative dot plots of MHC-II-positive cells in MI6 (**a**) and WTHER2 (**b**) tumours and frequency of MHC-II-positive cells in mice treated or not (NT) with trastuzumab by flow cytometry. *p*-values by unpaired *t*-test

To confirm that the change in MHC-II expression reflects the recruitment of immune cells in the tumour microenvironment by trastuzumab, we used 2 murine mammary carcinoma models available in our laboratory—MI6 and WTHER2—that overexpress human HER2 and are responsive and resistant to trastuzumab, respectively, when orthotopically implanted in syngeneic FVB mice.^20,16^ Tumours that were derived from the injection of MI6 in FVB mice, treated with 1 cycle of trastuzumab (1-week treatment) and analysed when their dimensions were not yet significantly different from untreated tumours (Figure S6), contained a higher proportion of MHC-II-positive cells than untreated tumours (Fig. 4a). On the contrary, trastuzumab did not induce this increase in resistant WTHER2 tumours (Fig. 4b and Figure S6). MHC-II-positive cells in MI6 tumours were nearly exclusively CD45+ cells (95 ± 1.7%), half of which were macrophages (CD11b+F4/80+: 52.5 ± 7.8%), independent of treatment, and one-third of which were CD11b−F4/80− cells (33.3 ± 5.5%) (Figure S7).

### Circulating biomarkers reflect the immunomodulatory activity of trastuzumab

**Fig. 5:**
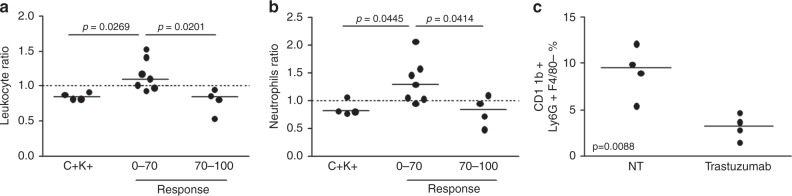
Modulation of circulating immune cells on trastuzumab treatment. **a**, **b** Modulation of leukocyte (**a**) and neutrophil (**b**) levels by trastuzumab in the blood of TRUP patients according to response to treatment. The ratio of cell counts between the time of Tru-cut biopsy and baseline in each patient is shown. *p*-values by unpaired *t*-test. **c** Percentage of neutrophils by flow cytometry, defined as Ly6G+F4/80− cells gated on CD11b+ cells, in the spleen of mice bearing MI6 tumours and treated or not (NT) with trastuzumab. *p*-value by unpaired *t*-test

Based on the ability of trastuzumab to recruit immune cells in the tumour microenvironment, specifically in patients who respond to trastuzumab-based therapy, we examined whether such immune modulation also occurred in the blood, analysing immune cell blood counts at baseline and at the time of Tru-cut biopsy. No differences in circulating immune cells before treatment were observed in patients by response (data not shown). Notably, the total leucocyte count was altered by trastuzumab (based on the post-treatment:pretreatment ratio) in responders compared with non-responders. Among leucocytes, neutrophil levels changed significantly by response. Specifically, neutrophil counts rose in non-responders, whereas clinical responders (C+K+) and patients in whom trastuzumab had the highest immunomodulatory activity experienced a decrease in circulating neutrophils at the Tru-cut biopsy compared with baseline (Fig. 5a, b). *ARG1*, an enzyme that is expressed primarily by neutrophils and M2 macrophages with immunosuppressive activity^21^ was significantly upregulated by trastuzumab in tumour tissue only in non-responders (Figure S8), suggesting that circulating neutrophil counts reflect the immunosuppressive status of the tumour microenvironment.

Accordingly, the proportion of neutrophils (CD11b+Ly6G+F4/80− cells) fell in the spleen of mice that harboured responsive MI6 tumours and were treated with trastuzumab compared with untreated animals (Fig. 5c and Figure S7), supporting the use of this marker as an indicator of trastuzumab activity.

## Discussion

By analysing samples of a window of opportunity trial that offered the possibility to dissect the activity of trastuzumab from that of chemotherapy, we observed that the efficacy of trastuzumab treatment is associated primarily with features of the immune system and that brief exposure to trastuzumab modifies the immune-related molecular properties of tumours, based on the response to treatment.

Although 1 cycle of trastuzumab reduced Ki67 positivity in nearly all tumours, Ki67 levels declined extensively primarily in tumours with a low DNA replication rate, high tyrosine kinase receptor activity, and high expression of extracellular matrix genes. These data are consistent with the positive association between low Ki67 positivity at baseline and the response to 3 cycles of trastuzumab alone, as reported by Mohsin et al.^22^.

In contrast, the size-reducing effect of trastuzumab was strictly associated with enrichment in immune genes, indeed tumours with high expression of immune genes at baseline were responsive to 1 cycle of trastuzumab. This finding is consistent with recent evidence that tumours with pre-existing high levels of immune cells or genes in their tumour microenvironment respond best to HER2-targeted therapies.^5^ The reduction in Ki67 levels by trastuzumab was independent of immune features, but only cases that achieved a C+K+ response benefited from the entire preoperative treatment, indicating that for trastuzumab to be efficacious, it must engage the immune system and block cell proliferation.

In certain cases, trastuzumab upregulated MHC-II immune genes, an increase that likely reflects the activation of the host immune system cycle triggering an antitumour immune response that could favour trastuzumab activity. These data are consistent with another study in which short-term exposure to trastuzumab upmodulated the CD4+ follicular helper T cell signature,^23^ considering that CD4+ T cells must recognise peptide-MHC-II complexes to become activated. The relevance of the trastuzumab-driven induction of immune infiltration in the response to treatment is strongly supported by the clustering analysis in which Tru-cut biopsies of responders are in the same cluster of C+K+ pretreatment samples, highly expressing immune genes.

Our gene expression and in situ analyses support the function of macrophages in the cytotoxic activity of trastuzumab, based on their ability to induce ADCP^7^ and engage the adaptive arm of the immune system. They act as antigen-presenting cells to CD4+ T cells, which are crucial for trastuzumab activity.^24^ The similar modulation of the LCK and HCK metagenes, reflecting the infiltration of T cells and monocytes,^13^ indicates that trastuzumab is active in tumours in which the entire immune system cycle is stimulated or inducible by trastuzumab. These results were confirmed in murine models, wherein we observed an increase in MHC-II positive cells after short-term trastuzumab treatment only in a mouse model that was responsive to trastuzumab—not in a trastuzumab-resistant model. Of note, increase of MHC-II positive cells within MI6 tumour microenvironment was observed before evidence of trastuzumab-induced tumour inhibition as occurred in patients responsive to complete preoperative trastuzumab-based therapy. Further studies are required to understand which signals are responsible for the trastuzumab-induced recruitment of MHC-II positive immune cells in the tumour microenvironment of responsive patients. One possible explanation derives from the well-known interaction between macrophages and apoptotic cells.^25^ In this context, as suggested by the enrichment of apoptosis pathway (Reactome) we observed in Tru-cut samples of responders vs non-responders (NES = 1.63, *p* < 0.0001), recruitment of MHC-II positive cells could derive from the induction of apoptotic cell death by trastuzumab that was described to be one mechanism of antibody activity.^22^

The lower levels of circulating neutrophils in patients who benefit from trastuzumab indicate the recruitment of these cells into the tumour microenvironment, where they likely participate in trastuzumab activity through their ability to mediate ADCC, as demonstrated in HER2-positive xenograft models.^26^ However, levels of circulating neutrophils may also inform about the activation status of patient immune system. Indeed, it is becoming evident that neutrophils can support tumour growth, based on the elevated counts of myeloid-derived suppressor cells (MDSCs) in the blood of patients with advanced cancer, reflecting tumour immune evasion.^27^ Whereas the reduction in circulating neutrophils in C+K+ tumours after trastuzumab treatment might be attributed to a decreased tumour burden due to the cytotoxic activity of trastuzumab,^28^ the differences between clinical responders and non-responders are unlikely to stem from disparities in tumour burden, because no differences in tumour dimension were appreciable at the time of the Tru-cut biopsy.

This pattern also holds for MI6 mice that were treated with or without trastuzumab. It is possible that these differences are attributed to the varying expansion of these cells, as induced by trastuzumab itself or through changes in the tumour cells. The significant negative correlation between circulating neutrophils at the diagnosis and the response to short-term trastuzumab favours the immune-suppressive activity of neutrophils in cancer patients, supporting the greater cytotoxic activity of trastuzumab in patients with low immune suppression.

Moreover, the lower basal expression of arginase in fast-responding tumours (C+K+) compared with other tumours, supports the rapid cytotoxic activity of trastuzumab in these tumours due to a favourable microenvironment, which is enriched in effector immune cells and has low immune suppression. Notably, patients who did not respond to treatment had higher circulating neutrophil and ARG1 levels in the tumour microenvironment at the time of Tru-cut biopsy, supporting greater immune suppression by trastuzumab, which is one explanation for its missed activity. Future research should determine the mechanism by which trastuzumab increases neutrophil levels in nonresponsive tumours to improve the activity of trastuzumab through immunotherapeutic strategies (e.g., anti-MDSC therapy) for patients who do not respond to treatment. Because the number of MDSCs modulates the activity of immune checkpoint inhibitors,^29^ evaluating the function of neutrophils in the response to trastuzumab could guide the development of new combinations of trastuzumab and anti-PD-L1 drugs, a promising approach for HER2+ tumours that has been demonstrated in preclinical models^30^ and in a trial of HER2+ BC patients.^31^

The limitations of this study include its small sample size and the lack of adequate tumour samples for all patients before treatment and after short-term exposure necessary to characterise immune infiltrates according to trastuzumab-response and to trastuzumab-induced apoptosis. Nevertheless, our data confirm the findings from preclinical studies on the mechanism of action of trastuzumab^5^ in human samples.

Our analyses demonstrate that short-term trastuzumab induces immune-related biomarkers that reflect the benefit from trastuzumab and activates the adaptive immune system, which has been crucial for the eradication of BC on treatment with trastuzumab in preclinical^24,30,32^ and clinical studies.^33^ The modulation of MHC-II by trastuzumab, primarily in patients with low basal immune infiltration, is consistent with our data on the change in NKG2D, a marker of NK and trastuzumab activity,^34^ suggesting that immune modulation by trastuzumab occurs only in patients in whom immune evasion has not been engaged.

This study, despite its exploratory nature, has strong translational relevance. Clinically it should prompt the examination of tissue-associated and circulating midcourse biomarkers of the efficacy of trastuzumab and compare them with basal pretreatment biomarkers in larger studies in which a window of exposure to anti-HER2 therapies without chemotherapy is incorporated into the trial design. The use of these biomarkers could become a valuable clinical approach for guiding patients toward the ideal regimen after brief exposure to trastuzumab. Moreover, in an era in which dual HER2 blockade has proven to be highly effective,^35,36^ this program would help identify patients who already benefit from single-agent trastuzumab with or without chemotherapy, supporting a de-escalation strategy.

## Electronic supplementary material

Supplementary figure legends

Figure S1

Figure S2

Figure S3

Figure S4

Figure S5

Figure S6

Figure S7

Figure S8

Table S1

Table S2

Table S1

## Availability of data and material

The gene expression profile data have been deposited and are available in the Gene Expression Omnibus repository (accession number GSE114082).
