# Supplementary figures and images for: Early immune modulation by single-agent trastuzumab as a marker of trastuzumab benefit
Source: Br J Cancer. 2018 Nov 27;119(12):1487–94. doi: 10.1038/s41416-018-0318-0 (PMC6288086; doi:10.1038/s41416-018-0318-0)

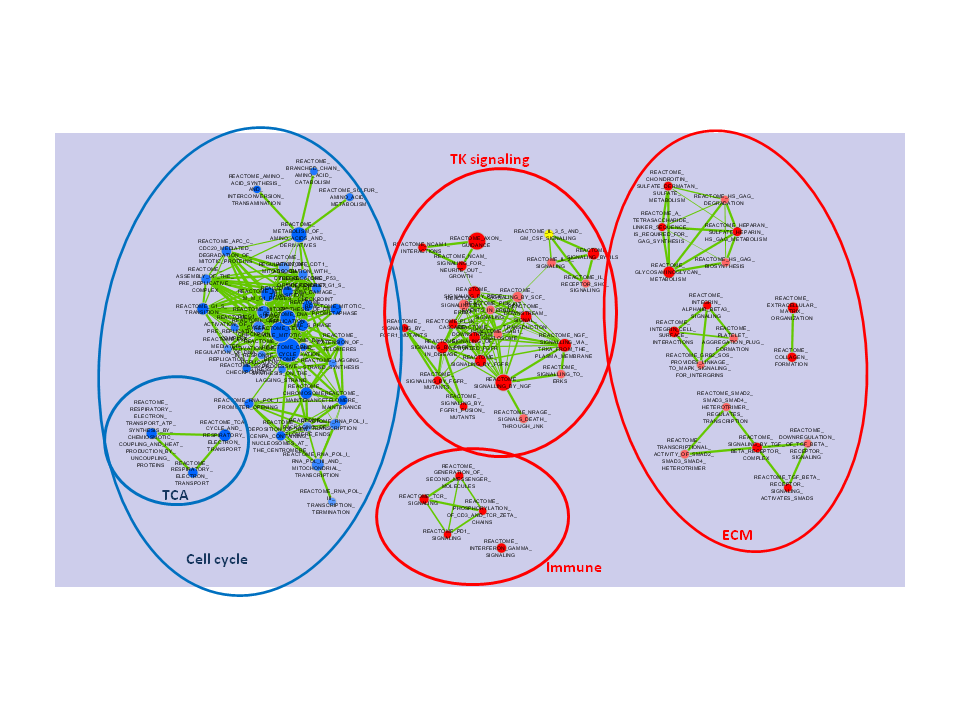

Supplement: Supplementary file 1 — Supplementary figure legends [file 41416_2018_318_MOESM1_ESM.tif]

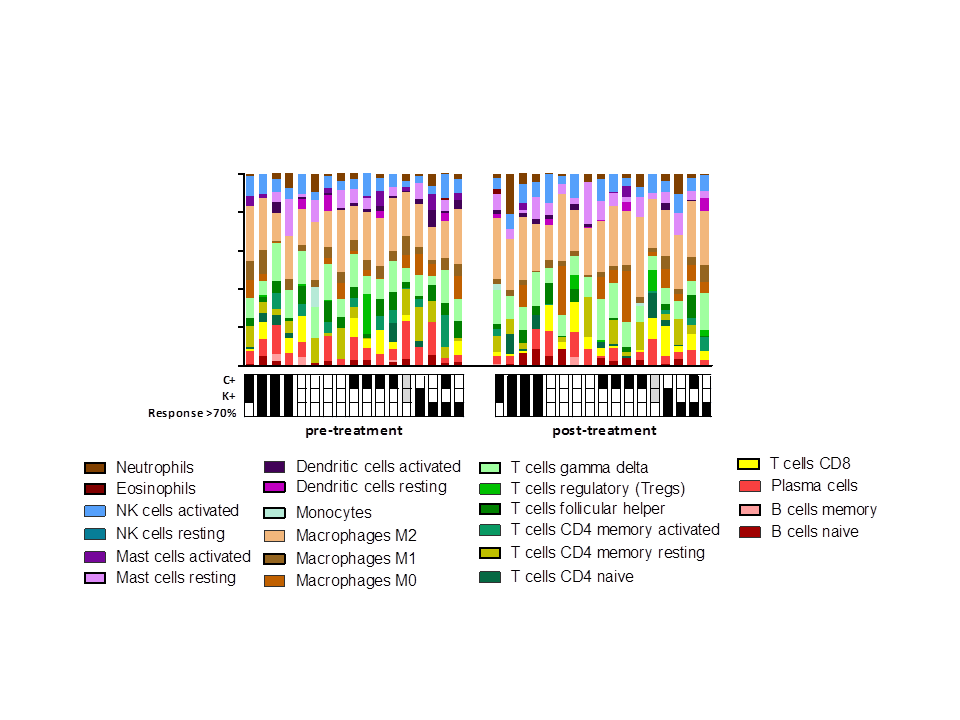

Supplement: Supplementary file 3 — Figure S2 [file 41416_2018_318_MOESM3_ESM.tif]

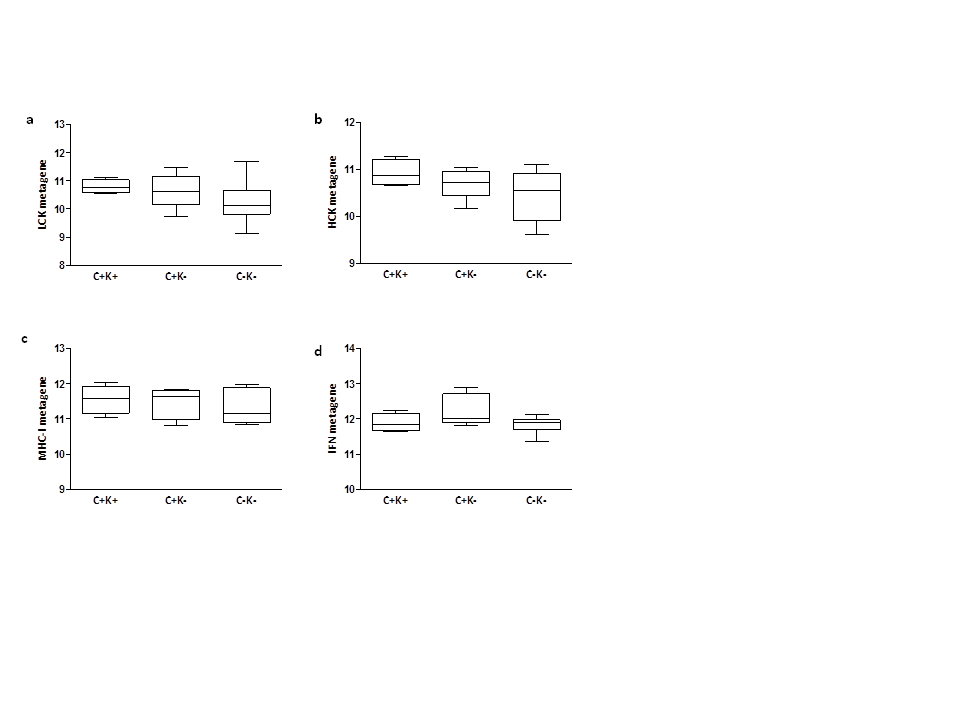

Supplement: Supplementary file 4 — Figure S3 [file 41416_2018_318_MOESM4_ESM.tif]

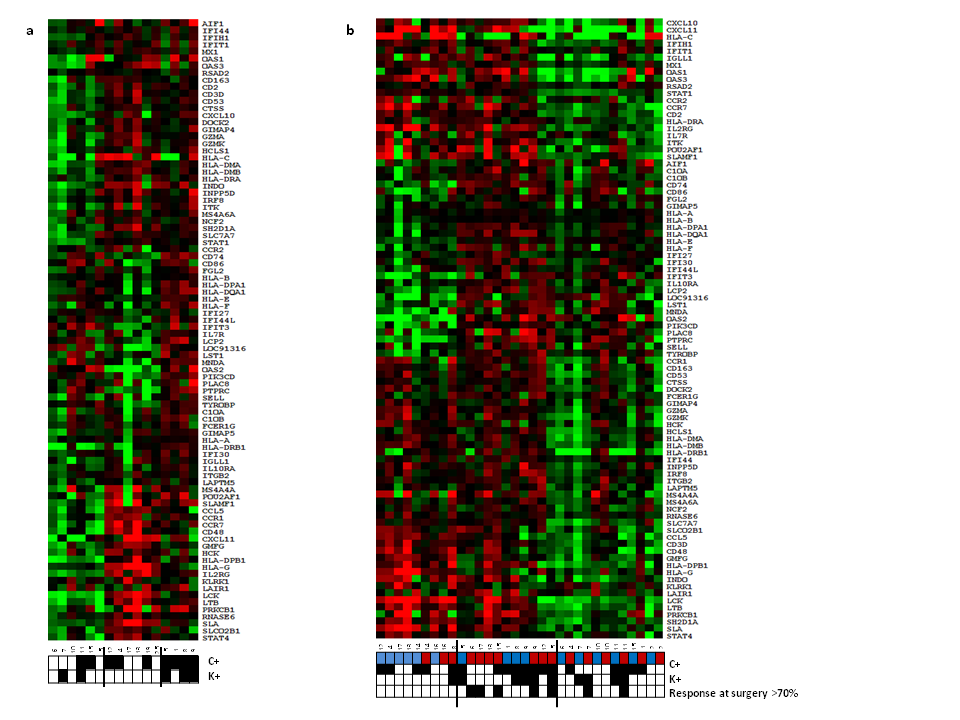

Supplement: Supplementary file 5 — Figure S4 [file 41416_2018_318_MOESM5_ESM.tif]

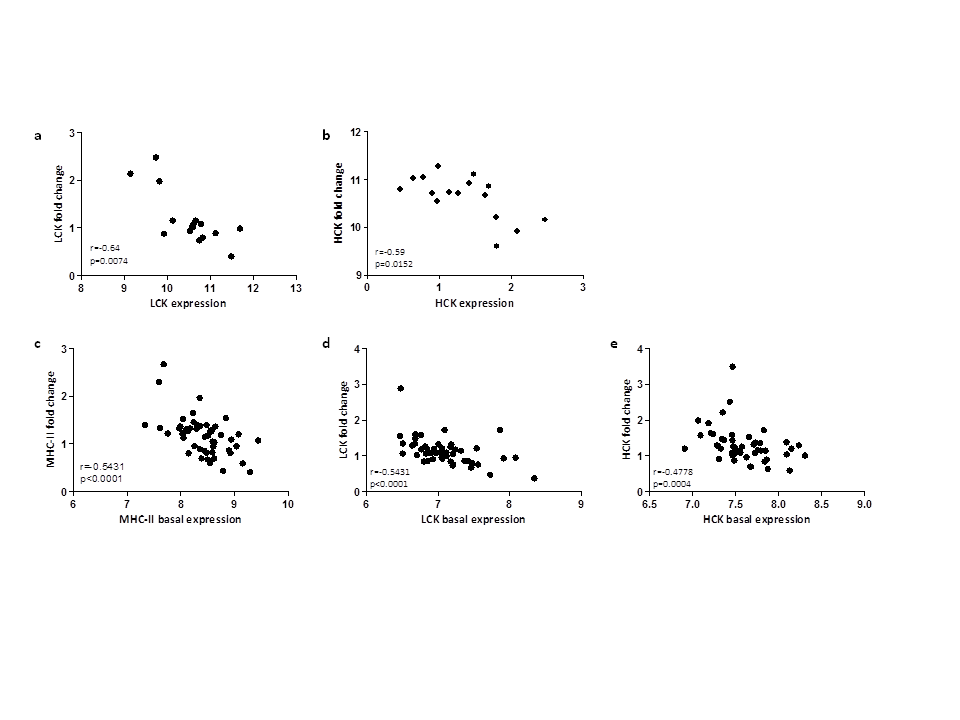

Supplement: Supplementary file 6 — Figure S5 [file 41416_2018_318_MOESM6_ESM.tif]

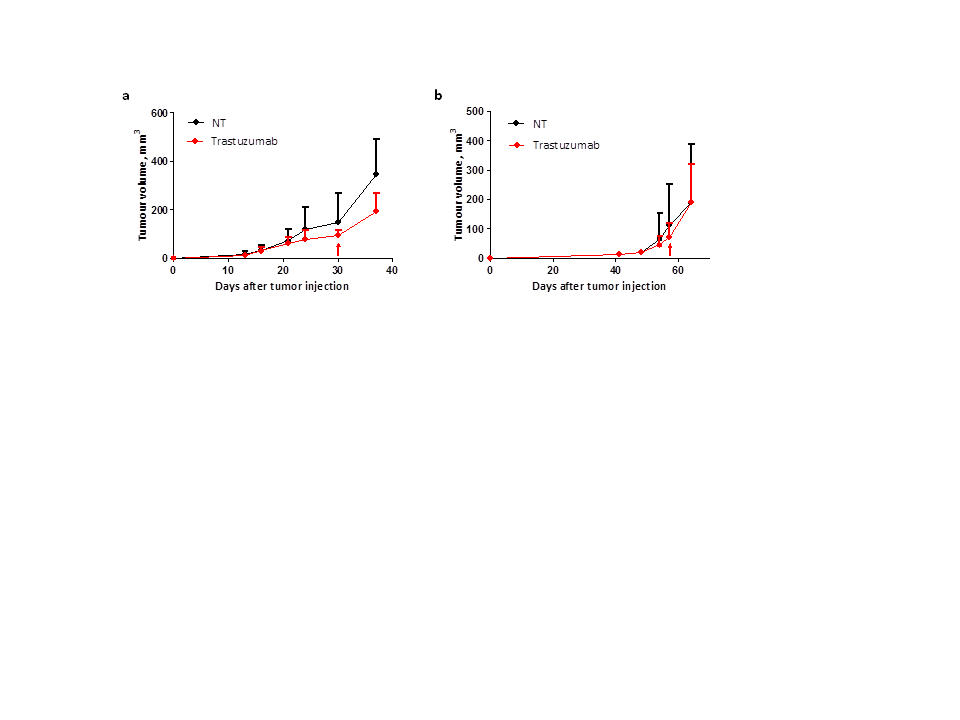

Supplement: Supplementary file 7 — Figure S6 [file 41416_2018_318_MOESM7_ESM.tif]

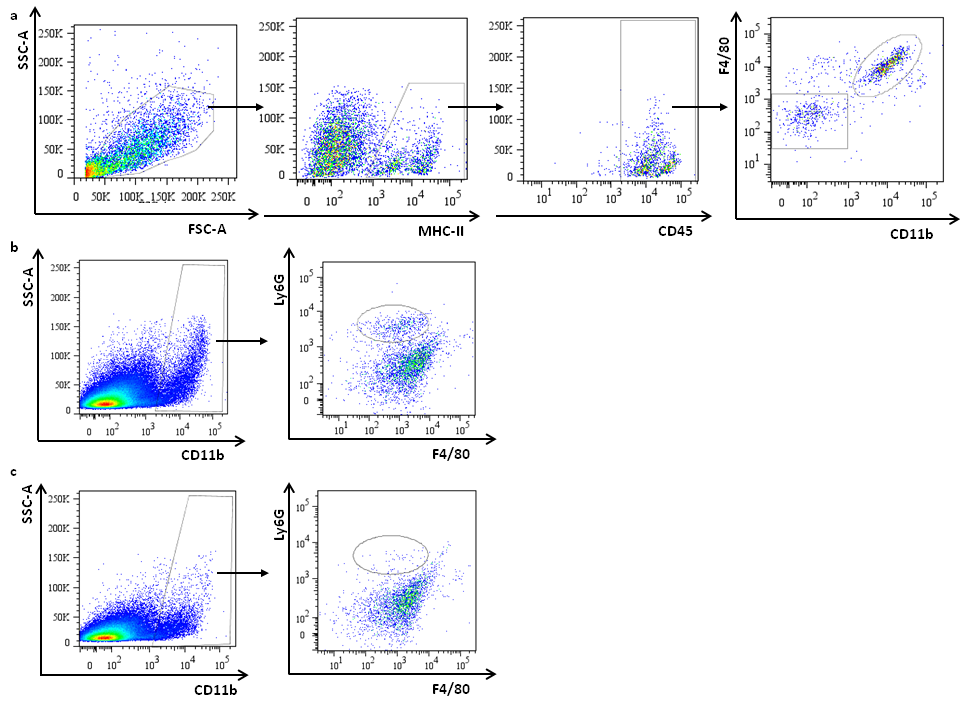

Supplement: Supplementary file 8 — Figure S7 [file 41416_2018_318_MOESM8_ESM.tif]
