# Supplementary material for: Early immune modulation by single-agent trastuzumab as a marker of trastuzumab benefit
Source: Br J Cancer. 2018 Nov 27;119(12):1487–94. doi: 10.1038/s41416-018-0318-0 (PMC6288086; doi:10.1038/s41416-018-0318-0)
Supplement: Supplementary file 9 — Figure S8 [file 41416_2018_318_MOESM9_ESM.docx]

**Table S1. Frequency of clinico-pathological characteristics of TRUP patients according to response at tru-cut and at surgery**

|  | **Response C**  **N (%)** | | **Response K**  **N (%)** | | **Response C and K**  **N (%)** | | | **Clinical response**  **N (%)** | |
| --- | --- | --- | --- | --- | --- | --- | --- | --- | --- |
|  | **C+, n=9** | **C-, n=7** | **K+, n=5** | **K-, n=11** | **C+K+, n=4** | **C+K-, n=5** | **C-K-, n=7** | **NR, (n=8)** | **R, (n=4)** |
| ***N pos*** | 5 (55) | 2 (40) | 3 (75) | 4 (40) | 1 (25) | 2 (40) | 2 (40) | 4 (50) | 0 (0) |
| ***T ≥3*** | 3 (33) | 4 (57) | 1 (20) | 6 (54) | 0 (0) | 3 (60) | 4 (57) | 3 (37) | 4 (100) |
| ***Grade III*** | 7 (78) | 4 (57) | 3 (60) | 8 (72) | 3 (75) | 4 (80) | 4 (57) | 5 (62) | 3 (75) |
| ***ER pos*** | 4 (44) | 5 (71) | 3 (60) | 6 (54) | 3 (75) | 1 (20) | 5 (71) | 3 (37) | 3 (75) |
| ***PGR pos*** | 3 (33) | 2 (29) | 1 (20) | 4 (36) | 1(25) | 2 (40) | 2 (29) | 2 (25) | 2 (50) |
| ***Ki67 >14%*** | 8 (89) | 6 (86) | 4 (80) | 10 (91) | 3 (75) | 5 (100) | 6 (86) | 7 (87) | 4 (100) |
| ***HER2-E*** | 3 (33) | 2 (29) | 2 (40) | 4 (36) | 1(25) | 2 (40) | 2 (29) | 1 (12) | 3 (75) |
| ***TRAR-low*** | 5 (55) | 4 (57) | 4 (80) | 9 (82) | 2 (50) | 3 (60) | 4 (57) | 3 (37) | 3 (75) |
