# Supplementary material for: Early immune modulation by single-agent trastuzumab as a marker of trastuzumab benefit
Source: Br J Cancer. 2018 Nov 27;119(12):1487–94. doi: 10.1038/s41416-018-0318-0 (PMC6288086; doi:10.1038/s41416-018-0318-0)
Supplement: Supplementary file 10 — Table S1 [file 41416_2018_318_MOESM10_ESM.docx]

**Table S2. List of pathways for each cluster significantly enriched in each comparison**

|  | **Pathway** | **C+ *vs* C-*** | **K+ *vs* K-*** | **C+K+ *vs* C+K-*** |
| --- | --- | --- | --- | --- |
| **IMMUNE** | REACTOME_PD1_SIGNALING | 2.26 | ns | 2.03 |
|  | REACTOME_INTERFERON_ALPHA_BETA_SIGNALING | 2.19 | ns | ns |
|  | REACTOME_PHOSPHORYLATION_OF_CD3_AND_TCR_ZETA_CHAINS | 1.98 | ns | 1.93 |
|  | REACTOME_IL3/5_AND_GMCSF_SIGNALING | ns | ns | 1.80 |
|  | REACTOME_IL_RECEPTOR_SHC_SIGNALING | ns | ns | 1.79 |
|  | REACTOME_IL2_SIGNALING | ns | ns | 1.71 |
|  | REACTOME_TCR_SIGNALING | ns | ns | 1.68 |
| **TCA** | REACTOME_PYRUVATE_METABOLISM | -1.95 | ns | ns |
|  | REACTOME_TCA_CYCLE_AND_RESPIRATORY_ELECTRON_TRANSPORT | ns | -1.72 | -1.65 |
|  | REACTOME_RESPIRATORY_ELECTRON_TRANSPORT | ns | -1.82 | -1.84 |
| **ECM** | REACTOME_COLLAGEN_FORMATION | ns | 1.82 | 2.16 |
|  | REACTOME_INTEGRIN_CELL_SURFACE_INTERACTIONS | ns | ns | 2.15 |
|  | REACTOME_EXTRACELLULAR_MATRIX_ORGANIZATION | ns | ns | 2.08 |
|  | REACTOME_NCAM1_INTERACTIONS | ns | 1.93 | 2.03 |
| **PROLIFERATION** | REACTOME_DNA_REPLICATION | ns | -1.96 | -2.09 |
|  | REACTOME_MITOTIC_M_M_G1_PHASES | ns | -1.97 | -2.05 |
|  | REACTOME_TELOMERE_MAINTENANCE | ns | -1.99 | -1.95 |
|  | REACTOME_CHROMOSOME_MAINTENANCE | ns | -1.88 | -1.89 |
|  | REACTOME_DEPOSITION_OF_NEW_CENPA_CONTAINING_NUCLEOSOMES_AT_THE_CENTROMERE | ns | -2.21 | -2.09 |
| **TK SIGNALING** | REACTOME_SIGNALLING_TO_ERKS | ns | 1.98 | 1.66 |
|  | REACTOME_SIGNALING_BY_PDGF | ns | 1.89 | 2.17 |
|  | REACTOME_PI3K_CASCADE | ns | ns | 1.68 |
|  | REACTOME_DOWNSTREAM_SIGNALING_OF_ACTIVATED_FGFR | ns | ns | 1.67 |
|  | REACTOME_GENERATION_OF_SECOND_MESSENGER_MOLECULES | ns | ns | 1.84 |

*NES (Normalized enrichment score) by GSEA. Ns: not significant
